# Glucose emission spectra through mid-infrared passive spectroscopic imaging of the wrist for non-invasive glucose sensing

**DOI:** 10.1038/s41598-022-25161-x

**Published:** 2022-11-29

**Authors:** Tomoya Kitazaki, Yusuke Morimoto, So Yamashita, Daichi Anabuki, Shiori Tahara, Akira Nishiyama, Kenji Wada, Ichiro Ishimaru

**Affiliations:** 1 Kagawa University, Faculty of Engineering and Design, Takamatsu, Kagawa Japan; 2 Kagawa University, Faculty of Medicine, Miki-cho, Kita-gun, Takamatsu, Kagawa Japan

**Keywords:** Infrared spectroscopy, Preventive medicine, Diabetes

## Abstract

Non-invasive blood glucose sensing can be achieved using mid-infrared spectroscopy, although no practical device based on this method has yet been developed. Here, we propose mid-infrared passive spectroscopic imaging for glucose measurements from a distance. Spectroscopic imaging of thermal radiation from the human body enabled, for the first time in the world, the detection of glucose-induced luminescence from a distance. In addition, glucose emission spectra of the wrist acquired at regular intervals up to 60 min showed that there was a strong correlation between the glucose emission intensity and blood glucose level measured using an invasive sensor. Thus, the new technology proposed here is expected to be applied to real-time monitoring of diabetic patients to detect hypoglycemic attacks during sleep and to detect hyperglycemia in a population. Moreover, this technology could lead to innovations that would make it possible to remotely measure a variety of substances.

## Introduction

With lifestyle changes, the number of people diagnosed with diabetes continues to increase, becoming a major problem worldwide^1^. Furthermore, in pre-diabetic subjects with metabolic syndrome and obesity, postprandial hyperglycemia is associated with increased cardiovascular events^2^. Therefore, real-time monitoring of glucose levels is highly desirable, but despite advancements made globally, a non-invasive technique has not yet been developed^3^.

Glucose sensors have been developed using a variety of approaches. For example, low-frequency reverse iontophoresis has been exploited to develop very thin devices attached to the skin, which must be worn throughout the day, to monitor glucose levels. The GlucoWatch Biographer, a device based on this method, was approved by the US Food and Drug Administration (FDA), but was discontinued in 2008 owing to problems with long warm-up time, skin irritation, and sweating. Since then, no device based on this method has appeared on the market^4–7^. Microwave-based glucose sensors are cost-effective and can be miniaturized. Importantly, they have excellent penetration depth, especially in the low frequency range. Skin reflection, transmission, and absorption are closely related to the dielectric properties or permittivity of the skin, which can be correlated with glucose variations. However, the dielectric constant is strongly affected by other blood components. Therefore, this method provides an indirect correlation between the dielectric constant and blood component changes and cannot directly detect changes in glucose^8,9^. There are numerous studies using near-infrared spectroscopy for biological measurements. However, methods using near-infrared spectroscopy generally require multivariate analyses to extract information about glucose from the overall spectrum. Therefore, an effective method with sufficient accuracy has not yet been established^3^. Other technologies have been widely investigated in recent years, although the resulting devices have yet to meet the requirements for practical applications. For example, devices with improved biocompatibility have been developed using 3D printing^10^, magnetohydrodynamics has been exploited to enhance the sensitivity of glucose detection^11^, and analyses have been improved by implementing neural networks^12^. In contrast, mid-infrared spectroscopy operates in the wavelength band known as the fingerprint region of a substance to provide molecule-specific spectra. Attenuated total reflection^13^ (ATR), high-power quantum cascade lasers^14^, and ytterbium-doped YAG lasers^15^ have been proposed to measure glucose levels in living organisms using mid-infrared spectroscopy. However, owing to its measurement principle, ATR can only measure to a shallow depth of approximately 5 μm from the surface, and light reaches only the stratum corneum in normal skin^16^. Therefore, the measurement site is limited to the oral mucosa^13^. Our proposed mid-infrared passive spectroscopic imaging method uses the synchrotron radiation of the substance as a spectroscope to obtain information from greater measurement depths (approximately 1.2 mm). Therefore, we believe that it is possible to measure glucose from a depth corresponding to the dermal layer of normal skin using mid-infrared spectroscopy, which is excellent for component identification.

The human body emits mid-infrared light, which can be detected using thermography to measure body temperature. Similarly, emission from the skin can be passively detected using mid-infrared passive spectroscopic imaging based on two-dimensional Fourier spectroscopy^17,18^, from which glucose-induced luminescence can be detected. Specifically, by equipping our original imaging-type two-dimensional Fourier spectrometer with a multi-slit, deterioration of interference definition between bright points is prevented^19^. This enables us to achieve a very high sensitivity that allows passive spectroscopic measurements of body temperature using an uncooled microbolometer array sensor. As a conventional approach to radiation measurements, point measurements of glucose values using radiation light via a band-pass filter have been proposed^20–22^. However, owing to the bandpass filters, light in a limited wavelength range is detected, and thus these methods have no flexibility in measurement. In addition, owing to the low measurement sensitivity, the measurement target is limited to tympanic membranes inside ear canals and materials inside sealed devices, which are not affected by ambient light. Importantly, our method has the potential to monitor multiple components, in addition to glucose, because it can acquire spectral characteristics in the wide sensitivity range of a microbolometer (8–14 µm). Moreover, our method is completely contactless. Here, we present the results of mid-infrared passive spectroscopic imaging of the wrist. We obtained time-series data, up to 60 min, for five subjects and cross-correlated the glucose emission intensity with the blood glucose level measured using an invasive sensor. We confirmed a high degree of correlation between the two parameters. This is the first feasibility demonstration of glucose monitoring using a completely contactless passive spectroscopic method.

## Experiments

When a substance is illuminated, the constituent molecules vibrate at their natural frequencies (wavelengths). Absorption spectroscopy uses these spectral characteristics to identify the components of a substance. In addition, materials also emit light at these wavelengths. In our proposed mid-infrared passive spectroscopic imaging, emission from the skin can be measured, and emission spectra can be obtained without preparing external light sources. In addition, emitted light from ambient heat sources (e.g., heat generated by people or machines) can be considered as spurious light generated by the measurement environment during the measurement. However, the proposed method is based on Fourier spectroscopy. Therefore, low-frequency fluctuations in radiance due to spurious light can be eliminated.

**Figure 1:**
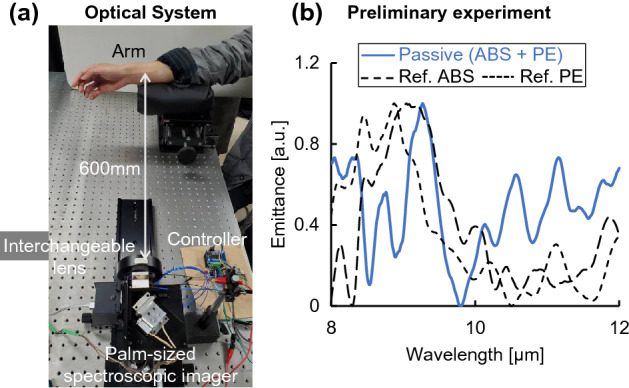
Optical system of mid-infrared passive spectroscopic imaging using subject A's wrist and back of the hand as measurement sites. **(a)** The optical system. **(b)** Preliminary experimental results using plastic (8-mm ABS plate and 1.20-mm PE thin film). The spectrum in (**b**) was normalized from 0 to 1.

Figure 1a shows the optical system, based on mid-infrared passive spectroscopic imaging, used for skin measurements. The optical system consisted of a spectrometer and one interchangeable lens (germanium, diameter = 50 mm, F = 0.5) in an infinite conjugate ratio design. Details of the internal optics of the spectrometer and the measurement principle are described in the “Methods” section. Because mid-infrared light is in the thermal band, it is considered to be affected by the synchrotron radiation inside the instrument. Therefore, the spectrometer was stabilized to within 1 °C using a heater during the measurement. First, we verified whether the proposed method could acquire emission spectra of skin surfaces. One adult male subject (over 18 years old) participated in the study. The wrist and back of the hand, selected as the measurement sites, were measured from a distance of 600 mm. The subject was seated and relaxed, and his arms were fixed in a stable position. The measurement distance of 600 mm was set to ensure that the skin area is covered by the measurement field of view. Although the measurement distance was fixed to 600 mm in this experiment, theoretically, the measurement performance of this spectrometer is independent of the distance. Because synchrotron radiation from the skin emits non-directionally, as the measurement distance increases, the areal density of light entering the spectrometer from a single emission point decreases, specifically the areal density is inversely proportional to the square of the distance. In contrast, the viewing area per pixel increases in proportion to the square of the distance. These two relationships cancel out the effect of distance on luminance, making the measurement independent of distance. In addition, the effect of gas (water vapor) in the optical path is not considered significant because the measurement bandwidth of the spectrometer is 8–14 µm, which is less sensitive to water vapor, as in thermography. As a preliminary experiment, we measured the emission spectrum of an acrylonitrile butadiene styrene (ABS) plate (8 mm in thickness) covered with a polyethylene (PE) thin film (1.20 mm in thickness, equivalent to the thickness of the skin) (surface temperature 30 °C) using the same optical system. The solid line in Fig. 1b shows the emission spectrum of this sample obtained using the proposed method, while the dashed line shows the reference spectrum for ABS, and the dotted line shows the reference spectrum for PE. The reference spectra are emission spectra of ABS (8 mm thick) and PE (1.20 mm) measured at a surface temperature of 30 °C. The reference spectra were compared with those in the spectral database (file name: 216_2018_1156_MOESM2_ESM.xlsx) provided by Primpke et al.^23^ and the waveforms were confirmed to match (shown in Supplementary Fig. S1). The emission spectrum was the sum of the ABS and PE spectra, confirming that the proposed method detected emissions from a depth of approximately 1.20 mm or greater. Furthermore, as shown in Supplementary Fig. S2, there was a strong correlation between the average emission intensity of the characteristic peak of ABS at 9.0–9.5 µm and the thickness of the PE thin film, which was varied in steps from 0.24 to 1.20 mm. However, the results of this preliminary experiment are qualitative. The information obtained from any depth depends on the absorbance of the material in the upper layer. Although not verifiable using human skin, this experiment suggests the possibility of measuring glucose concentration in the dermis. The results demonstrating the glucose detection capability of the proposed method are also shown in Supplementary Fig. S3. Measurements of glucose solutions with different concentrations (0.08–100%) showed a strong concentration dependence of the glucose emission intensity. Therefore, these experiments strongly suggests that emission from a depth of approximately 1.2 mm and glucose levels equivalent to blood glucose concentrations can be detected.

Next, we validated the proposed method for quantitative glucose detection. In this experiment, the wrists of five subjects (four men and one woman, demographics of the subjects are shown in “Methods”) were measured using both the proposed method and an invasive blood glucose sensor (GLUCOCARD PlusCare, Arkray Corporation, Kyoto, Japan) every 10 min from 0 min (start of measurement) to 60 min (end of measurement). For each measurement, the proposed method was used four times and the invasive blood glucose sensor was used once. In addition, subjects drank a commercially available drink. The sugar content of this beverage is 6,200 mg/dL (20,500 mg in 330 mL) with sucrose as the main ingredient (Tully’s Coffee Honey Milk Latte, 330 mL, ITO EN, LTD., Tokyo, Japan). Subjects drank this drink after the 10-min measurement to increase their blood glucose levels. In other words, the 0- and 10-min data were treated as pre-meal data, and the 20- to 60-min data as post-meal data.

## Results and discussion

**Figure 2:**
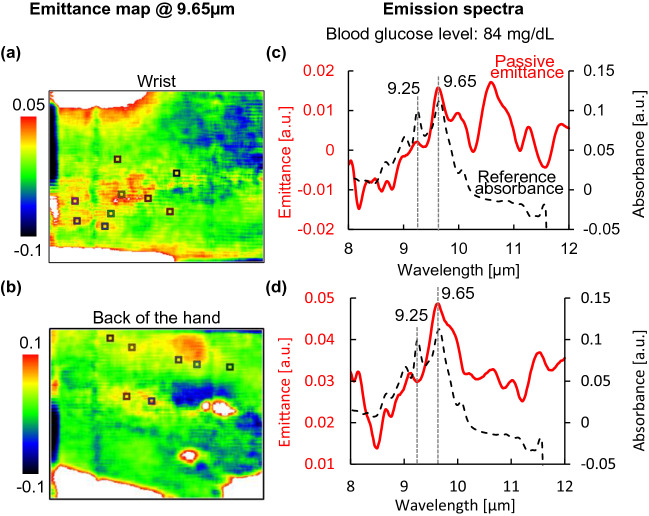
Results of mid-infrared passive spectroscopic imaging using subject A's wrist and back of the hand as measurement sites. **(a)** Spectral emission map at 9.65 µm of the wrist. **(b)** Spectral emission map at 9.65 µm of the back of the hand. **(c)** Emission spectrum of the wrist. **(d)** Emission spectrum of the back of the hand. The ranges of the color maps in (**a,b**) were adjusted manually. The red solid line in (**c,d**) shows the emission spectrum obtained using the proposed method, and the black dashed line shows the absorption spectrum of glucose solution measured using FTIR.

The data obtained using the proposed method were processed to produce pseudo-color maps. The blood glucose level of subject A at the time of measurement was 84 mg/dL. The absorption spectrum of the aqueous glucose solution measured using a Fourier transform infrared spectrophotometer (FTIR), which is conventionally used in mid-infrared spectroscopy, is shown in Fig. 2c,d as a black dashed line. In this graph, it can be seen that the representative peaks of glucose in the measurement band of the spectrometer are at 9.25 and 9.65 µm. Therefore, emittance at the wavelength of 9.65 µm was assigned to glucose emission (Fig. 2a,b), and high glucose emission was observed in a band around the center of the wrist and on the back of the hand. Although only visually confirmed, blood vessels were visible at the same locations, suggesting that glucose concentrations were high in the surrounding areas. Figure 2c,d shows the averaged emission spectra by selecting the coordinates with high emission in the pseudo color maps (square marker positions). Currently, it is necessary to artificially select areas of high emittance, but in the future, this step will be automatic. A comparison of these two spectra with the FTIR spectrum of a glucose solution showed that the emission peaks corresponding to glucose were observed at wavelengths of 9.25 and 9.65 µm. This result demonstrates that the proposed method can be used to measure the effect of glucose variations. In addition, by imaging in two dimensions, high emissions can be obtained in the region around blood vessels.

**Figure 3:**
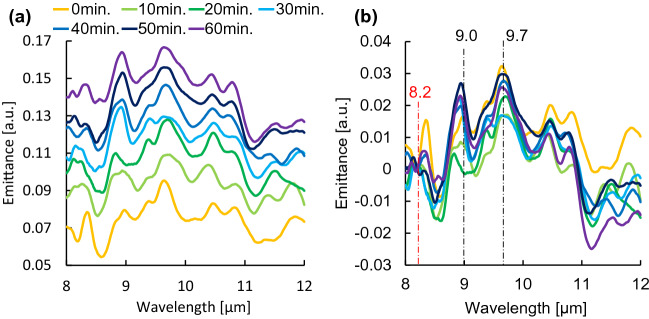
Temporal changes in the emission spectrum of subject A. **(a)** Emission spectrum of subject A taken every 10 min. **(b)** Emission spectra of subject A normalized to the intensity of a glucose-independent peak (8.2 µm).

**Figure 4:**
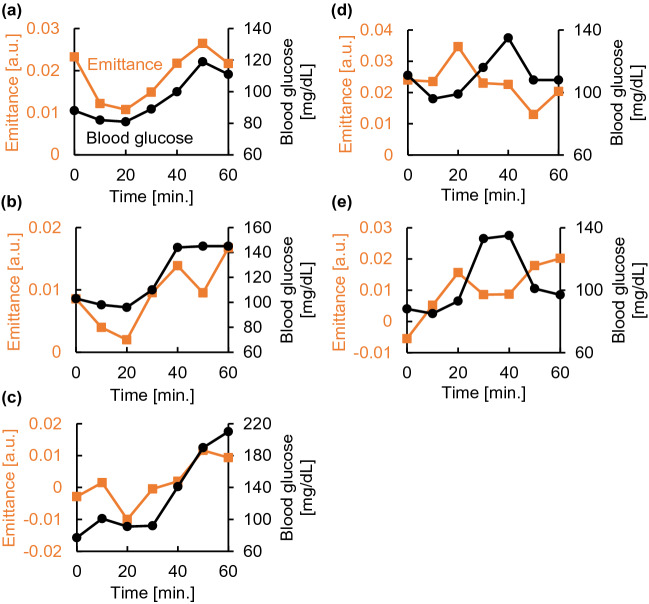
Temporal changes in emission and blood glucose levels based on periodic measurements taken every 10 min. **(a)** Time trends of emission and blood glucose levels of subject A. **(b)** Time trends of emission and blood glucose levels of subject B. **(c)** Time trends of emission and blood glucose levels of subject C. **(d)** Time trends of emission and blood glucose levels of subject D. **(e)** Time trends of emission and blood glucose levels of subject E. The emittance in (**a–e**) are the average emissions at 9.0 and 9.7 µm, corresponding to the glucose emission peaks.

**Figure 5:**
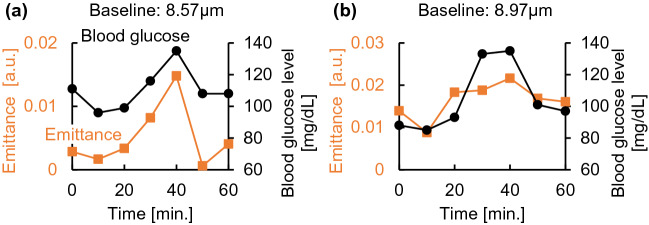
Optimizing the correction wavelength in the measurements of subjects D and E. **(a)** Results for subject D (corrected wavelength of 8.57 µm). **(b)** Result for subject E (corrected wavelength 8.97 µm).

**Figure 6:**
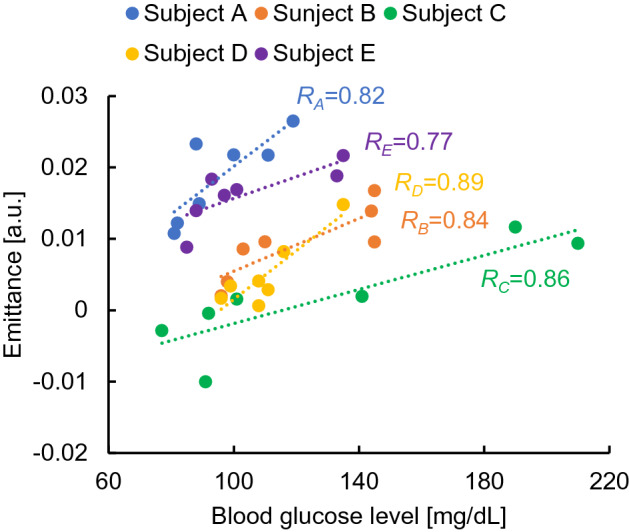
Cross-correlation evaluation of emittance and glucose levels for subjects A to E.

Figure 3a shows the radiation spectrum of subject A at each time interval. The glucose emission peak was successfully detected in the spectrum at each time point. However, the baseline of the emittance spectrum increased with time owing to a decrease in the skin surface temperature during the measurement. Therefore, as shown in Fig. 3b, all spectra were normalized by assigning the emission level of 0 to the peak at 8.2 µm, a peak with low glucose dependence. Owing to the principle of Fourier spectroscopy, the peak wavelengths may shift depending on the resolution of the instrument. In this analysis, 9.0 and 9.7 µm were set as the optimum values for the glucose peaks, and the average luminosities at 9.0 and 9.7 µm were compared with the blood glucose level as the glucose signal. For the radiation spectra of subjects B to E, the peak intensities were similarly normalized, and the time trends of the average emittance of the glucose peaks at 9.0 and 9.7 µm and blood glucose levels are shown in Fig. 4a–e. The results of subjects A, B, and C showed that the average emission intensity at 8.2 µm was directly proportional to the effect of glucose variations. In contrast, this trend was not observed in the results of subjects D and E, which likely originated in factors such as skin adhesions, perspiration, and reflected light during the measurement. Therefore, we investigated the reference wavelengths at which the correlation with the value of the invasive blood glucose sensor was high in the results of subjects D and E by a total number search. The results of subjects D and E after the investigation are shown in Fig. 5. The average luminous intensity was directly proportional to the blood glucose level by setting the reference wavelength to 8.57 µm for subject D and to 8.97 µm for subject E. As shown in Fig. 6, calibration curves were prepared for each subject, and a high correlation was obtained. However, there were differences in the initial deviation of emittance and responsiveness among subjects.

**Figure 7:**
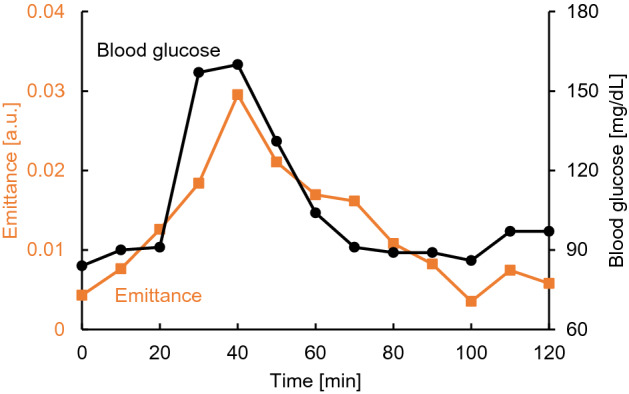
Time course of emittance and blood glucose levels during the additional experiment for subject A.

To investigate the relationship between the emittance and physiological signs of the subjects, an additional experiment was conducted with the cooperation of subject A. The measurement period was extended to 120 min, and physiological signs (corneal water content, body temperature, blood pressure, heartbeats, and skin surface temperature) that could be collected without changing the body position were recorded every 10 min. The measurement results are shown in Fig. 7. The measurement results were normalized with the wavelength of 8.2 µm as the reference wavelength. As shown in Fig. 7, a high correlation was obtained between blood glucose level and emittance. In addition, when the blood glucose level decreased after the subject consumed the beverage, the emittance also decreased and returned to the baseline. As shown in Supplementary Fig. S4, the emittance was not affected by the recorded corneal water content, body temperature, blood pressure, and heart rate. Therefore, it can be assumed that changes in glucose concentration can be detected without interference from changes in the physiological signs recorded in this study. However, a high dependence of emittance intensity at the reference wavelength of 8.2 µm on skin surface temperature was observed, as shown in Supplementary Fig. S5. This was due to the temperature dependence of the multi-slit^19^ built into the spectrometer, which improves the interference definition.

## Prospects for glucose measurements by mid-infrared passive spectroscopic imaging

Here, we demonstrate our proposed mid-infrared passive spectroscopic imaging for remote glucose monitoring. In experiments with five subjects, a high correlation between blood glucose and emittance was obtained by correcting for baseline variations caused by changes in the skin surface temperature, which occurred through a mechanical mechanism, as shown in Supplementary Fig. S5. Thus, we were able to demonstrate the feasibility of remote glucose monitoring using this method. However, several issues remain unresolved.

First, it is necessary to clarify the causes of initial deviations in emittance and differences in responsiveness between subjects. The effects of physiological signs during measurements and measurement conditions, such as adhesions on the subject’s skin surface, perspiration, and reflected light, should be considered. At this stage, we have confirmed that physiological signs, namely keratin moisture content, body temperature, blood pressure, and heartbeats, do not affect the emittance. Other parameters should be evaluated.

The next step is to examine the effect of changes in the subject’s body position. The change in body position is largely influenced by the subject’s movement during the measurement. The subject’s movements may cause noise. The current measurement system does not have a tracking function to follow the subject’s movements. Therefore, the subject is asked to remain still during the measurement period. By introducing tracking technology used in image processing, we believe that glucose measurements will be possible as long as the subject is within the angle of view during the measurement.

The next step is to examine calibration strategies for the final user-oriented system. As shown in Fig. 6, the calibration curves have different slopes for different subjects at the current stage of development. Therefore, we believe that if the system is put into practical use, it will be necessary to simultaneously measure blood glucose levels with an invasive blood glucose sensor for the first few times to create a calibration curve tailored to each individual subject. We plan to collect data to evaluate the persistence of the calibration curve in the future. Optimistically, however, non-contact thermometers based on thermal imaging cameras, which are widely used, do not require calibration. Mid-infrared passive spectroscopic imaging is a spectrometer version of thermography. Thermography estimates body temperature from the emitted light, which is the integral of the emitted light at 8 to 14 µm. Our instrument measures this emitted light spectroscopically. The main component of the emitted light from the skin is water, which has a broad emission spectrum in the mid-infrared region, providing a baseline for the emission spectrum of a living body. Human water content is considered to be in the range of 60–70% and does not vary greatly among individuals. Therefore, the glucose level is estimated from the emissivity due to glucose, which changes on the stable baseline, and the influence of individual differences on the stable baseline is considered to be negligibly small. We believe that further improvement of the performance of the device will make calibration-free measurements possible.

Finally, to improve the accuracy of the measured effect of glucose variations, four measurements were taken at each time point. As shown in Supplementary Fig. S6, that the average emittance varied by ± 0.01, likely causes by temperature instability of the spectrometer and arm movement. Thus, improving the temperature control of the spectrometer and compensating for the aforementioned arm movements should mitigate the variation in emittance. In addition, interstitial glucose generally responds to changes in blood glucose levels with a delay, and that the two have different magnitudes and different rates of change^24^. Because this method can detect emissions from a depth of approximately 1.2 mm, more accurate measurements can be achieved by identifying which emission depends on the change in blood glucose and which is related to interstitial glucose and then examining a correction model.

To realize remote glucose measurement by mid-infrared passive spectroscopic imaging, we will extend our experiments to include a greater number of subjects and accumulate data from measurements of subjects other than the human body for further validation. In particular, the effects of electrolytes, proteins, and water as well as physiological signs and potential ambient noise on the depth of measurements need to be systematically and thoroughly investigated.

## Conclusion

The present study demonstrated that the effect of glucose variations could be measured remotely and noninvasively using a mid-infrared passive spectroscopic imaging technique based on two-dimensional Fourier spectroscopy. We succeeded for the first time in the world in detecting the effect of glucose variations in the infrared spectrum of the human body measured passively using a very simple and inexpensive device, as presented herein. Time-course radiation spectra of five subjects confirmed a clear correlation between blood glucose levels and the intensities of specific emission peaks, suggesting the usefulness of the system for monitoring the effect of glucose variations. In contrast to other methods, the proposed method is completely non-contact and can measure the effect of glucose variations in two dimensions, and we aim to realize a system that can automatically measure the effect of glucose variations simply by living within the spectrometer’s angle of view. This is expected to be developed into a stationary technology that can simultaneously measure the effect of glucose variations of multiple people and enable the identification of diabetic patients in a group. Furthermore, it is expected to contribute to safety measures, such as monitoring hypoglycemic attacks in diabetic patients during sleep. Further research will lead to the realization of non-invasive glucose sensors by improving measurement accuracy and minimizing the effects of individual differences. In the future, this technology is expected to lead to a completely new concept for diagnostic imaging in that all vital signs, in addition to the effect of glucose variations, can be monitored simply by observing a human being from an overhead vantage.

## Methods

### Internal optical system of an imaging-type two-dimensional Fourier spectrometer

The spectrometer, with external dimensions of 105 mm × 90 mm × 50 mm (width × depth × height) and a mass of 1.25 kg, is so small and lightweight that it can be placed in the palm of the hand. The spectrometer includes a near-common-pass phase-shift interferometer. Inside the spectrometer, the objective lens (germanium, lens diameter: 25 mm, focal length: 15 mm) and imaging lens (germanium, lens diameter: 25 mm, focal length: 15 mm) are placed such that their optical axes are orthogonal. A phase-variable filter comprising a fixed mirror and a movable mirror driven by a piezoelectric element is placed at the intersection of the optical axes. The detector is an uncooled microbolometer infrared area sensor (array size: 320 × 256 pixels, pixel pitch: 12 µm, Noise Equivalent Temperature Difference (NETD): 50 mK, sensitivity range: 7.5–13.5 µm, Boson 320, FLIR Systems Inc., Wilsonville, OR, USA). A multi-split plate (aperture: 36 µm, shading: 24 µm) is set in the conjugate plane of the detector to prevent the cancellation of bright spots and to improve the interference definition. The changeable lens has an F value of 0.5 (germanium, lens diameter: 50 mm, focal length: 25 mm).

### Selection of subjects

The subjects’ vital signs (body temperature, blood pressure, heart rate, respiratory rate, body water content, and keratin moisture content) were measured and confirmed to be within the values of healthy subjects. Five subjects in their 20 s (four males and one female) from the laboratory participated in this study. Subjects were 21 ± 1 years old, and they had a body temperature of 36.6 ± 0.3 °C, heartbeats of 70 ± 11 bpm, maximum blood pressure of 114.5 ± 14.5 mmHg, minimum blood pressure of 69 ± 6 mmHg, body moisture content of 49.3 ± 7.7%, keratin moisture content of 42 ± 4%, and respiratory rate of 17.5 ± 3.5 bpm. The body temperature was measured with an electronic thermometer (MC-687, Omron Healthcare Co., Ltd., Kyoto, Japan). The blood pressure was measured with an upper arm sphygmomanometer (HCR-7201, Omron Healthcare Co., Ltd., Kyoto, Japan). The body water content was measured with a body composition analyzer (RD-917L, Tanita Corporation, Tokyo, Japan). The keratin moisture content was measured with a capacitance-based keratin moisture meter (MoistSense, Moritex Corporation, Saitama, Japan). The heart rate was determined by counting the pulse at the subject’s wrist, and the respiratory rate was counted from the subject’s chest movements. All procedures performed in this study and informed consent forms were reviewed and approved by the Ethics Committee of Kagawa University (#2020-162) and were consistent with the 1964 Declaration of Helsinki and its later amendments or comparable ethical standards. Written informed consent was obtained from all the participants prior to the study.

### Method of calculating emittance

The subject’s surface temperature was measured using thermocouples (306 Data Logger Thermometer, Center Technology Corp., New Taipei City, Taiwan) during the measurement. The emission spectrum of the subject’s skin was divided by the emission spectrum of a blackbody at this temperature, thus eliminating the blackbody radiation curve from the spectrum.

### Principle of the imaging-type two-dimensional Fourier transform spectroscopy

WE developed an original optical system, called the near-common path phase shift interferometer. This system is highly robust against mechanical vibration and can be configured as a very simple optical system without adding a mechanism to prevent mechanical vibration. An object plane can be modeled as a collection of bright spots that optically emit rays of light non-directionally. For example, focusing on a single bright spot, a group of non-directionally emitted light rays is converted into a collimated light beam by an objective lens and focused onto the image plane by an imaging lens to form an optically conjugate bright spot image. The object’s surface is covered with numerous bright spots, and the bright spots at different positions form conjugate bright spot images at different positions on the imaging plane by focusing the light at different angles, i.e., in different directions of the angle of view. These conjugate bright point images form a two-dimensional image, which is the geometric optical view of the image formation phenomenon. In contrast, from the wave optics point of view, a group of rays emitted from one bright spot can be considered to form a bright spot as an interference phenomenon when multiple rays of light reach the image-forming plane in a phase-aligned state and interfere with each other.

We introduced a phase-variable filter to provide an arbitrary optical path length difference to half of this parallel object light beam. Initially, the phases of all the rays are aligned on the imaging plane, and the interference conditions are such that they strengthen each other. Then, a half-wavelength optical path length difference is applied to half of the object light beam by the phase-variable filter. In this case, the light rays in the upper half and the light rays in the lower half weaken each other under the interference condition, and thus the bright spot image darkens. If a half wavelength, or one wavelength in total, is applied, the phase of the upper and lower halves returns to the initial state, and the conjugate bright spot image becomes brighter because the phase of the upper and lower halves of the ray group returns to the initial state, and thus the conditions for constructive interference are restored. Thus, depending on the optical path length difference given by the phase-variable filter, the conjugate bright spot image will periodically brighten and darken repeatedly. In spectroscopy, we are dealing with the light of multiple wavelengths. For example, for a component with a long wavelength, the period of the interference intensity change is longer because the optical path length difference per wavelength is longer. Additionally, the shorter wavelength component will have interference intensity changes at a higher frequency. The sum of these different periodic interference intensity changes is observed in a single pixel. This multi-wavelength interference intensity change is called an interferogram.

Because the interferogram is composed of multiple periodic cosine waves, the spectral characteristics, i.e., the intensity at each frequency, or relative intensity at each wavelength, can be obtained analytically by using the mathematical Fourier transform.

### Improvement of interference definition by introducing a multi-slit

The object plane can be optically modeled as a collection of bright spots^19^. Each bright spot forms an airy pattern on the imaging plane. These countless airy patterns on the object plane are considered aggregates of pairs of airy patterns satisfying the Rayleigh criterion. Two airy patterns satisfy the Rayleigh criterion when the center of an airy disk and the first dark ring of a neighboring airy pattern are tangent to each other. With the operation of phase shift, the luminance value of the center of the Airy disk changes periodically from light to dark in terms of interference luminance value. However, the first dark ring is shifted out of phase by π, and the interference luminance value changes periodically from dark to light. Therefore, in the two airy patterns satisfying the Rayleigh criterion, the central luminance value of the airy disk and the luminance value of the first dark ring cancel each other out. Therefore, when the spatial frequency of the object surface texture is low, an interferogram with high sharpness cannot be obtained because the interference intensity changes between the bright spots cancel each other out. Therefore, we installed a multi-slit on the conjugate surface formed by the exchange lens to thin out the bright spots based on the Rayleigh criterion^19^. In this multi-slit, apertures and shaded areas with a width approximating the spatial resolution are periodically arranged. In the present system, the multi-slit is placed on the conjugate plane with 3 pixels for the aperture and 2 pixels for the shading, using infrared-absorbing glass as the base material. This made it possible to acquire interferograms with a high degree of clarity even for measurement targets, such as the arm, of which the surface texture has a low spatial frequency in the infrared region. Thus, mid-infrared passive spectroscopic imaging is now possible because interferograms with a high degree of clarity can be obtained even with weak synchrotron radiation emitted from the body.

## Supplementary Information

Supplementary Figures.

## Data Availability

The datasets used and/or analysed during the current study available from the corresponding author on reasonable request.
