# Supplementary material for: Glucose emission spectra through mid-infrared passive spectroscopic imaging of the wrist for non-invasive glucose sensing
Source: Sci Rep. 2022 Nov 29;12:20558. doi: 10.1038/s41598-022-25161-x (PMC9708671; doi:10.1038/s41598-022-25161-x)
Supplement: Supplementary file 1 — Supplementary Figures. [file 41598_2022_25161_MOESM1_ESM.pdf]

Glucose emission spectra through mid-infrared passive spectroscopic imaging of the wrist for non-invasive glucose sensing

Tomoya Kitazaki[1], Yusuke Morimoto[1], So Yamashita[1], Daichi Anabuki[1], Shiori Tahara[1], Akira Nishiyama[2], Kenji Wada[2], and Ichiro Ishimaru[1*],

1 Kagawa University, Faculty of Engineering and Design, Takamatsu-City, Kagawa, Japan

2 Kagawa University, Faculty of Medicine, Miki-cho, Kita-gun, Kagawa, Japan

Email: ishimaru.ichiro@kagawa-u.ac.jp

Supplementary Information

Supplementary Figure legends

Supplementary Fig. S1| Comparison of emission spectra of ABS (a) and PE (b) measured using mid-infrared passive spectroscopic imaging with absorption spectra in the database.

Supplementary Fig. S2| Evaluation of the effects of varying the PE thin film thickness from 0.24 mm to 1.20 mm in steps. **(a)** Emission spectra at each thickness. **(b)** Cross-correlation between the average emission intensity of the characteristic emission peak of ABS at 9.0–9.5 μm and the thickness of the PE thin film.

Supplementary Fig. S3| Measurements of glucose solutions at different concentrations using mid-infrared passive spectroscopic imaging. **(a)** Emission spectra by concentration. The emittance of the powder is indicated on the second vertical axis. **(b)** Evaluation of the correlation between emittance and concentration below 1% (1,000 mg/dL) concentration.

Supplementary Fig. S4| Changes in physiological signs and emittance during an additional experiment for subject A. Temporal changes in **(a)** keratin moisture content, **(b)** blood pressure, **(c)** body temperature, and **(d)** heartbeats.

Supplementary Fig. S5| Evaluation of changes in skin surface temperature and baseline emittance during the additional experiment for subject A. **(a)** Skin surface temperature and baseline emittance over time. **(b)** Cross-correlation between skin surface temperature and baseline emittance.

Supplementary Fig. S6| Variation in emittance for four repeated measurements in the additional experiment for subject A.

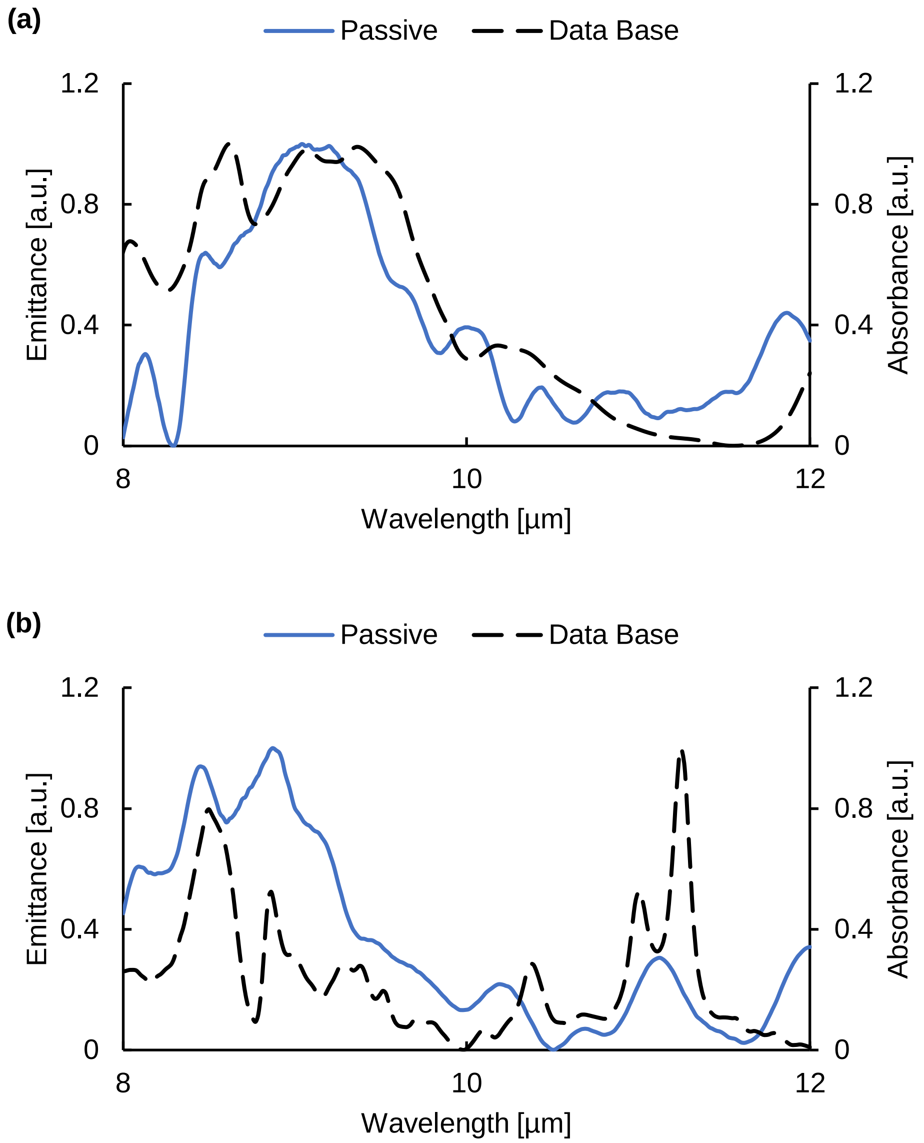

Supplementary Fig. S1| Comparison of emission spectra of ABS (a) and PE (b) measured using mid-infrared passive spectroscopic imaging with absorption spectra in the database.

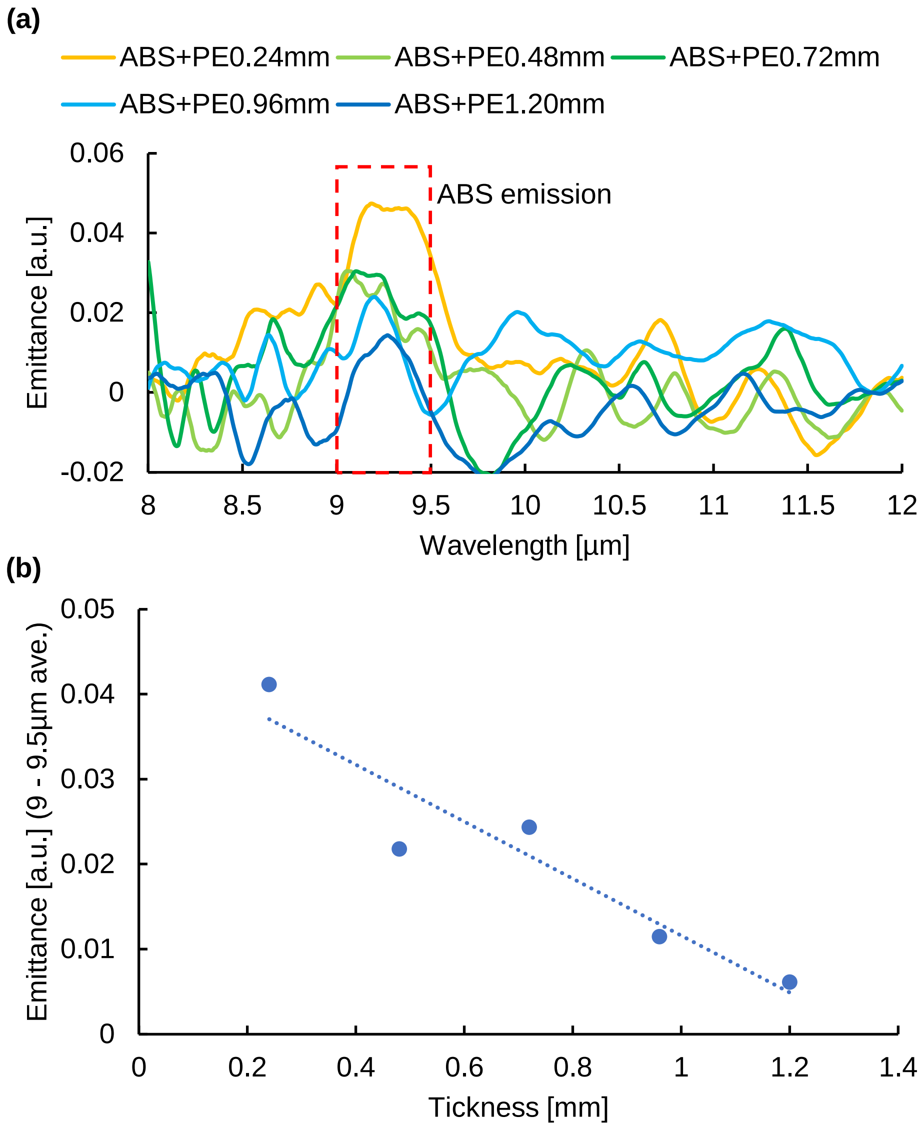

Supplementary Fig. S2| Evaluation of the effects of varying the PE thin film thickness from 0.24 mm to 1.20 mm in steps. (a) Emission spectra at each thickness. **(b)** Cross-correlation between the average emission intensity of the characteristic emission peak of ABS at 9.0–9.5 µm and the thickness of the PE thin film.

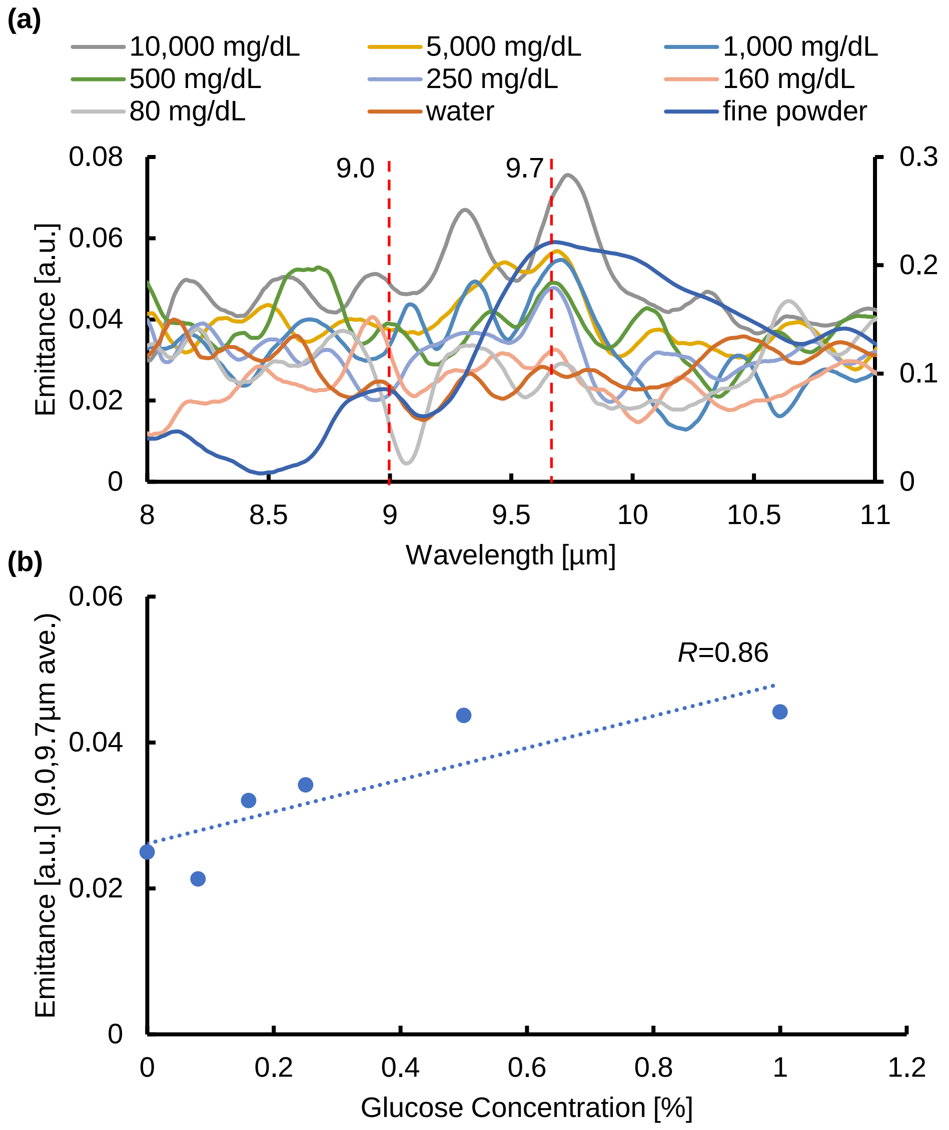

Supplementary Fig. S3| Measurements of glucose solutions at different concentrations using mid-infrared passive spectroscopic imaging. (a) Emission spectra by concentration. The emittance of the powder is indicated on the second vertical axis. **(b)** Evaluation of the correlation between emittance and concentration below 1% (1,000 mg/dL) concentration.

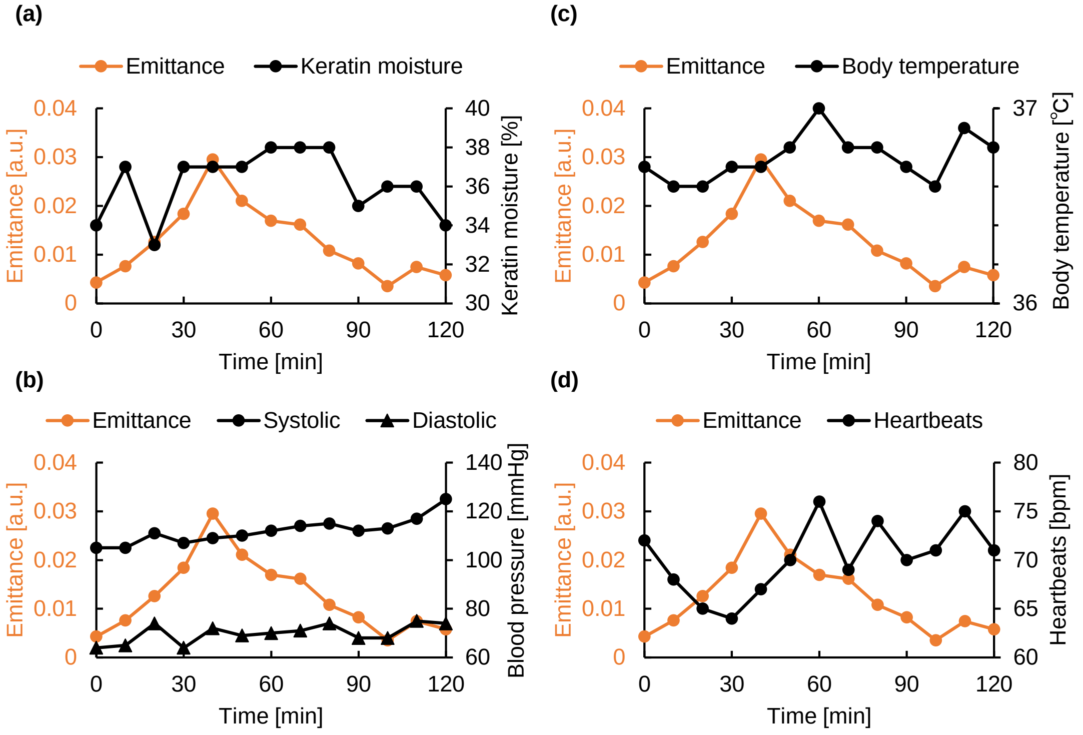

Supplementary Fig. S4| Changes in physiological signs and emittance during an additional experiment for subject A. Temporal changes in **(a)** keratin moisture content, **(b)** blood pressure, **(c)** body temperature, and **(d)** heartbeats.

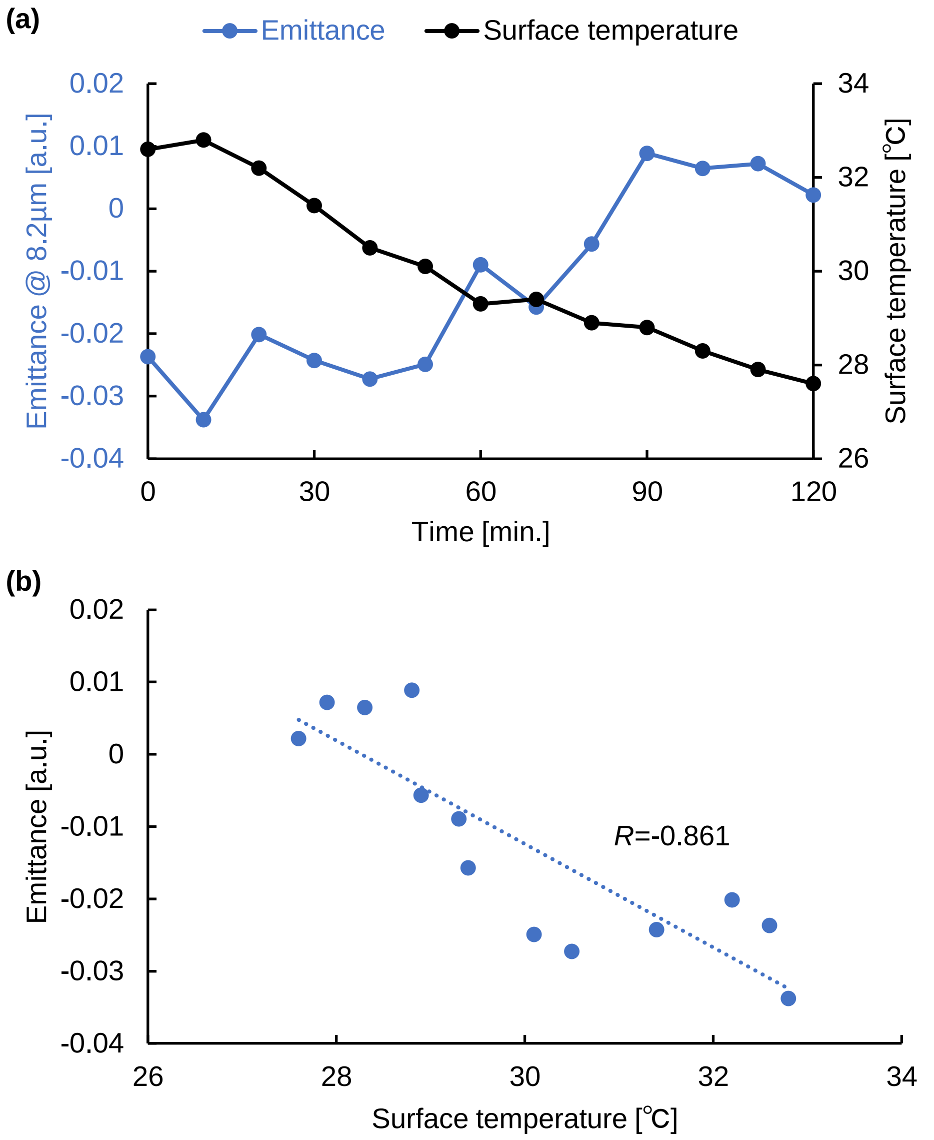

Supplementary Fig. S5| Evaluation of changes in skin surface temperature and baseline emittance during the additional experiment for subject A. (a) Skin surface temperature and baseline emittance over time. **(b)** Cross-correlation between skin surface temperature and baseline emittance.

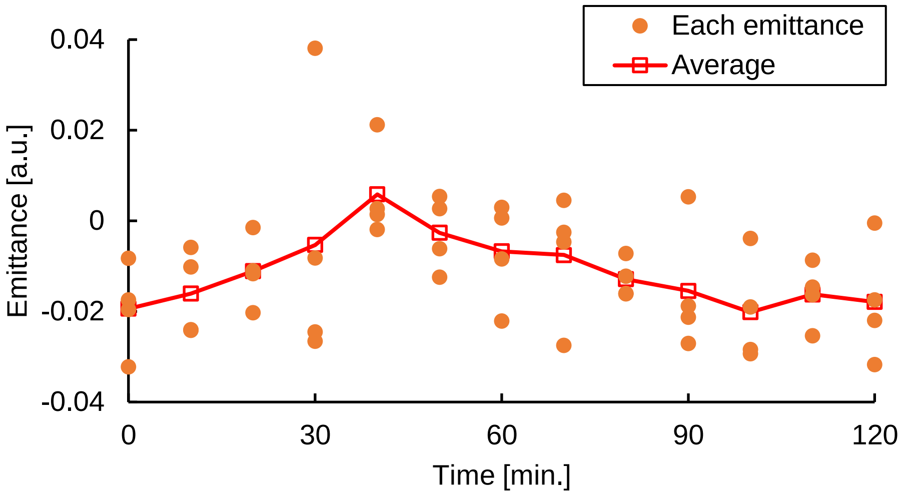

Supplementary Fig. S6| Variation in emittance for four repeated measurements in the additional experiment for subject A.
